# A Review of Clinical Applications and Side Effects of Methotrexate in Ophthalmology

**DOI:** 10.1155/2020/1537689

**Published:** 2020-08-11

**Authors:** Ge Wang, Xiaoyan Peng

**Affiliations:** Beijing Institute of Ophthalmology, Beijing Tongren Eye Center, Beijing Tongren Hospital, Capital Medical University, Beijing Ophthalmology and Visual Science Key Lab, Beijing, China

## Abstract

Methotrexate (MTX) is a folate analog widely used against a range of diseases including malignancies and autoimmune disorders. Its high effectiveness-price ratio also won extensive application in ophthalmology. On the other hand, although MTX has an excellent pharmacological efficacy, MTX associated side effects in clinical use, which vary from patient to patient, are nonnegligible. There is no comparatively systematic review on MTX associated side effects and its risk factors. This review aimed to reveal novel clinical approaches of MTX and its adverse effects in order to provide a reference for ophthalmic scholars in clinical application of MTX.

## 1. Introduction

**Figure 1:**
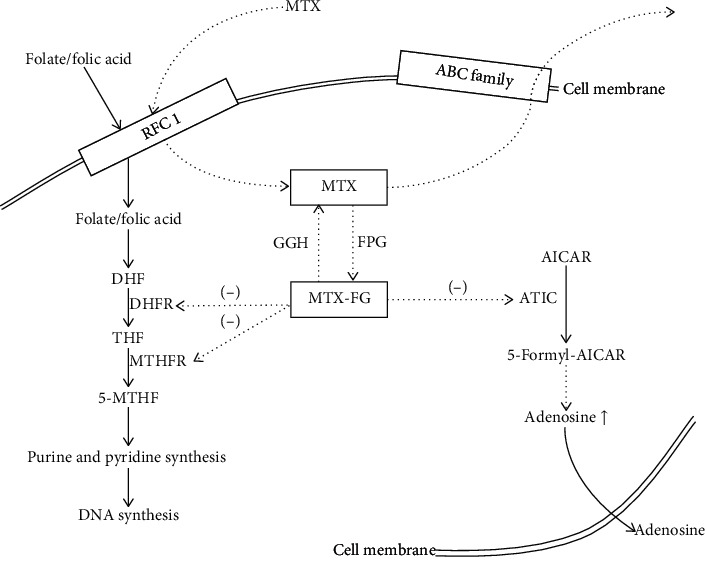
The cellular pathway of folate and MTX. Dietary folate enters the cells through RFC1, as well as MTX. In low-dose MTX treatment, MTX inhibits enzymes of the folate pathway. Ultimately, MTX leads to an increase in intracellular adenosine level, which would cause anti-inflammatory effects. RFC-1 = reduced folate carrier 1; ABC family = adenosine triphosphate-binding cassette (ABC) family; DHF = dihydrofolate; THF = tetrahydrofolate; GGH = *γ*-glutamyl hydrolase; FPG = folylpolyglutamate synthase; MTX-PG = methotrexate polyglutamate; DHFR = dihydrofolate reductase; MTHFR = methylene tetrahydrofolate reductase; AICAR = aminoimidazole carboxamide ribonucleotide; ATIC = AICAR transformylase.

Methotrexate (MTX) is an antifolate metabolite that inhibits DNA synthesis, repair, and cellular replication. It was firstly used as one of the essential treatments of pediatric leukemia [1, 2]. According to previous studies, MTX has also been used to treat rheumatoid arthritis (RA) and psoriasis as anti-inflammatory and immunomodulatory agent [3], as MTX could not only optimize the efficacy of biological disease modifying antirheumatic drugs (DMARDs) [4, 5], but also make the therapeutic goals via lower doses in comparison with other conventional synthetic DMARDs [5].  Figure 1 shows the pathway of folate in DNA synthesis, the cellular pathway of MTX, and how MTX works inside the cell. While immediate and low-dose MTX is used to treat nonmalignant and immune-mediated disorders, high-dose MTX (HD-MTX, more than 500 mg/m^2^/week) is widely used to treat malignancies. Until now, HD-MTX (with or without radiation therapy) is still the backbone of most modern chemotherapy regimens [6], as well as the prevention of systemic/central nervous system (CNS) lymphoma recurrence at a dose of 3 g/m^2^ per week [7].

MTX has also been widely applied in ophthalmic diseases, systemically and locally. Recently published articles pay more attention to new clinical applications, routes of administration, and newly discovered side effects, which are foci of this review.

## 2. Clinical Applications in Ophthalmology

As one of the known corticosteroids-sparing agents, MTX has been widely used in the treatment of anterior, intermediate, posterior, or pan uveitis; scleritis; and ocular mucous membrane pemphigoid [8], as well as advanced proliferative diabetic retinopathy [9]. However, followed researches reveal that MTX works with a significant difference in effectiveness ratings by anatomic location of inflammation [10], with treatment success achieved most commonly in patients with anterior uveitis and scleritis [11]. In the treatment of noninfectious intraocular inflammation, oral and intravenous are the most common routes with a usual dose range of 7.5 mg to 25 mg weekly. The typical dose observed was 12.5 mg/week [12, 13], which is in the range of low-dose MTX. The median time to achieve the success of treatment, defined as control of inflammation with the ability to taper corticosteroids to 10 mg or less daily, ranges from 4.5 months to 9 months for MTX [14, 15].

Intravitreal MTX injection, with or without systemic chemotherapy and radiotherapy, has already been used to treat primary intraocular lymphoma patients [7, 16–19]. According to Larkin et al. [20], intravitreal MTX injection could achieve remission in a proportion of patients with primary intraocular lymphoma. What is particularly noteworthy is that although MTX has a slow rate of onset of effect, when it was used to treat intraocular lymphoma via intravitreal injection, it prolonged local remission of ocular disease even with an aggressively growing tumor [16]. Therefore, it has been taken as a relatively first-line choice for the treatment of recurrent intraocular lymphoma [17], although the treatment for primary intraocular lymphoma is lacking solid justification because of the limited retrospective and prospective case series [21]. Local treatment via intraocular injection provides a consistent therapeutic MTX concentration to reduce the systemic MTX associated side effects [19]. Therefore, intraocular MTX injection is worth trying, especially for unilateral ocular diseases.

## 3. New Approaches of Applications of MTX

### 3.1. MTX Used against Epithelial Downgrowth

Previous studies have already demonstrated safety of intravitreal MTX [18]. It has been used to treat intraocular lymphoma and proliferative vitreoretinopathy because of its antiproliferative properties [22]. There is a novel use of intravitreal MTX for recurrent epithelial downgrowth which was not treated by surgical and medical methods. Lambert et al. [23] administered intravitreal MTX to patients with refractory proliferative membrane after cataract surgery, while membrane peel and endolaser treatment failed. The injection of MTX was administered alone, based on previous protocols and the presumed half-life of drugs in vitreous cavity. After 12 injections totally, there was no membrane recurrence. This case suggests that intravitreal MTX plays a role in treatment against epithelial downgrowth.

### 3.2. MTX Used in Conventional Therapy-Resistant Diseases

Generally, the antivascular endothelial growth factor (anti-VEGF) therapy has dramatically improved the prognosis of neovascular age-related macular degeneration (nAMD). However, there are still some patients who remain refractory to anti-VEGF therapy, which is termed as treatment-resistant nAMD. As there is evidence that MTX has effects in interrupting the angiogenesis cascade at various levels [24], Kurup et al. offered intravitreal MTX to patients who were refractive to standard anti-VEGF therapy [25]. Although it was an off-label use, the patients' visual acuity improved at follow-up visit, while ophthalmic imaging examinations showed significantly reduced cystoid macular edema. Thus, patients who are refractory to traditional anti-VEGF therapy might benefit from intravitreal injection of MTX.

This approach is not alone. Khalil et al. [26] had 400 *μ*g/0.1 ml of MTX intravitreal injection once monthly administrated to 20 adult Behcet's disease (BD) patients suffering from BD-associated ocular inflammation with posterior segment involvement. Their results prove that intravitreal MTX improves visual acuity, reduces posterior segment manifestations associated with Behcet's disease, and allows the reduction of corticosteroids and immunosuppressive drugs [26]. These results also supported Taylor and associates who conducted trials on 15 patients with unilateral uveitis and/or cystoid macular edema [27]. Their clinical trials suggest that intravitreal MTX may help patients with uveitis-associated posterior segment involvement to regain normal anatomical structure and then allowed the reduction of immunosuppressive therapy.

## 4. The Pharmacogenetics of MTX

With molecular sequencing and high-throughput technology, large numbers of genetic polymorphisms can now be detected accurately and rapidly [28]. Researchers pay more attention to pharmacogenetics, the study of genetic polymorphisms in drug-metabolizing enzymes and the translation of inherited differences to differences in drug effects [29]. The genes encoding transporting proteins and metabolizing enzymes for MTX are also known to harbor functionally significant SNPs. The SNPs may influence the efficacy of MTX and have been suggested as potential risk factors for enhanced MTX toxicity, even in low-dose regimens, based on previous researches [30].

The research of pharmacogenetics of MTX could be divided into genetic polymorphisms affecting MTX transport and SNPs that influence enzymes in the cellular pathway of MTX [29].

Once taken, MTX enters the cell through an active transport which is mediated by the reduced folate carrier 1 (RFC1). The loss of RFC1 gene expression might lead to effects of uptake and intracellular levels of MTX. A G80A SNP of RFC1 was proposed [31] making a decreasing [32] or increasing [33, 34] effect on intracellular level of MTX. Therefore, a significant association between RFC1 SNPs and MTX toxicity should be considered. Chango et al. state that these SNPs strongly impact the overall MTX associated side effects by resulting in altered cellular MTX concentration, but with no influence on MTX efficacy [35]. However, some researchers argue that these SNPs have no definite effect [36]. Thus, it remains controversial whether SNPs of RFC1 affect the transport of MTX. Moreover, P-glycoprotein, a membrane transporter that has influences on the disposition and bioavailability of MTX [37], was studied. SNPs of ABCB1, including C3435T SNP and C1236T SNP, were believed to have effects on the expression of P-glycoprotein [38]. Gervasini et al. speculate the C1236T SNP of ABCB1 affects the administered doses of MTX and the incidence of hematological toxicity [28]. However, just like G80A SNP, there are disputes about the influences of these SNPs, as different studies had different outcomes [29].

Metabolizing enzymes were also being analyzed, given the critical role of transporters in disposition of MTX and its active products, as well as the folate metabolism. MTX pharmacogenetics mostly focused on the SNPs in the MTHFR gene. The present study shows that genetic polymorphisms in the folate metabolic pathway and in MTX transporters influence the toxicity but not the efficacy of the low-dose MTX treatment in patients with autoimmune diseases [39]. For example, C677T and A1298C are known in MTHFR gene to result in a lower enzyme activity [39]. Windsor and associates reported that MTHFR A1298C and C677T were associated with MTX related nephrotoxicity and anemia [40]. These SNPs might be associated with decreased activity of methylenetetrahydrofolate reductase, elevated plasma homocysteine levels, and altered distribution of folate [41]. Thus patients with this genotype were more vulnerable to potential MTX induced toxicity since these reactions above may lead to slower folate metabolism and slower cell repair [42]. Weisman et al. used univariate logistic regression to reveal that the MTHFR C677T also increases the occurrence of side effects in central nervous system, manifested as headache and lethargy [43]. However, Lambrecht et al. argued that MTHFR C667T was not a predictive factor for toxicity [42]. Berkani et al. found no association between A1298C polymorphism and MTX toxicity [44]. Interestingly, Grabar et al. claimed that the patients with MTHFR 1298C genotype have a lower risk for MTX toxicity than the carriers of MTHFR 1298A allele [39].

To date, the study of pharmacogenetics of MTX continues. An increasing number of SNPs have been found to be possibly associated with the efficacy and toxicity of MTX. The newly discovered genotypes include C347G in ATIC and 5′-UTR 28-bp repeat and 3′-UTR 6-bp deletion in TYMS, which may influence both efficacy and toxicity of MTX; similarly, factors that may affect MTX associated toxicity are, for example, A2756G in MS and A66G in MTRR [29, 39, 45, 46]. The genes and their SNPs that might be associated with the effects and side effects of MTX are summarized in Table 1. Growing evidences suggest that a single genetic factor is unlikely to adequately predict the efficacy and toxicity of MTX in polygenic disease, such as RA and autoimmune associated ocular disease. Given the impact of MTX in several metabolic pathways, a complex of multiple risk genotypes examination would help to predict the efficacy of MTX and to identify patients who may have adverse effects from MTX administration.

Taken together, the efficacy and toxicity of MTX may remain associated with the genetic markers in the patients. Therefore, although this remains a controversial subject, it is reasonable to believe that pharmacogenetics may be able to predict who is at risk of MTX associated adverse effects and may help in maximizing the benefit-risk ratio of MTX.

## 5. The Side Effects of MTX

The dose-limiting toxicity of MTX mainly includes hepatotoxicity and nephrotoxicity [56–59], but mortality has often been reported due to either pneumonitis or secondary infections [60].

Some experts divided MTX associated pulmonary complications into inflammatory, infectious, and lymphoproliferative [61]. In the authors' opinion, all MTX related side effects can be classified into these three categories according to the pharmacological effects of MTX.

Major adverse events for MTX are related to the folate antagonism and primarily affect highly proliferative tissues such as bone marrow and gastrointestinal mucosa [62]. Given the immunosuppression effect of MTX, pancytopenia was one of the most frequent severe toxicities of methotrexate [30]. Meanwhile, the risk of developing an infectious process is increased all along the treatment, and the severity of the infected disease would be worsen [63, 64], including common bacterial infections, herpes zoster eruptions, and opportunistic infections. According to previous studies, the risk is larger than that with other disease modifying antirheumatic nonbiological drugs (DMARDs).

Secondly, the MTX acts as the hapten [65] and is likely to react directly with nucleophilic groups present in proteins, i.e., to combine with endogenous protein [66, 67]. The protein adducts thus act as an antigenic signal to direct the effector arm of the immune response [68]. The provoked immune responses are most commonly type I (immediate hypersensitivity) and type III (immune complex) reactions [66]. Hypersensitivity pneumonitis is the most common, severe, and unpredictable complication, with a mortality of up to almost 25% [69].

Moreover, a few studies have shown that long-term MTX use can lead to lymphoproliferative disorders (LPDs) in both nodal sites and extra nodal sites, such as the skin, lungs, epipharynx, thyroid gland, nasal cavity, spleen, and kidneys, especially for patients who are positive for EBV infection [70–73]. The reported frequency of EBV positive in MTX associated LPDs patients is 27%–50% [74]. Although the mechanism of onset is not fully understood, it is believed that the combination of immunodeficiency and the immunosuppressive effect of MTX has been implicated in the pathogenesis of MTX associated LPDs. The World Health Organization (WHO) has classified MTX associated LPDs as lymphoid neoplasms, whether iatrogenic or immunodeficiency associated diseases [73, 75]. MTX associated LPDs often take a spontaneous remission, which tends to complete mostly within 4 weeks, after the discontinuation of MTX [74]. But there are a few reports showing that the lymphoid neoplasms occur even after stopping using MTX [76].

### 5.1. The Effects of Administration Routes

Generally, the side effects of MTX depend on the route of administration. Dose-dependent [77] gastrointestinal side effects are the most frequent side events with orally administered MTX, as oral administration is the most common delivery method [56, 57, 77, 78]. More than 90% of MTX is excreted by the renal system; thus MTX associated nephrotoxicity is common among patients taking MTX. Fortunately, the resolution usually occurs after discontinuation of therapy and salvage treatment with high-dose corticosteroids [79]. Therefore, to achieve treatment with less side effects, the appropriate route of administration and dose of MTX are necessary. During the treatment, monitoring of patients' general condition matters.

Adverse effects of intravitreal injections of MTX occur only within the eye, including hyperemia, keratopathy, cataract, iridocyclitis, vitreous hemorrhage, retinal detachment, maculopathy, and endophthalmitis [80].

Splitting doses of MTX, rather than intravenous administration, is a new attempt to avoid MTX associated side effects. MTX is split and given twice or thrice in a week to achieve higher bioavailability and better clinical response [81, 82], thus providing us with a novel method of oral administration of MTX with less adverse effects.

### 5.2. Is Low-Dose MTX Safer?

Based on clinical cases observation, side effects which can lead to discontinuation of MTX are rare during the typical ophthalmology treatment because of the lower dose of MTX required [30]. The application of low-dose MTX regimen has also become one of the main therapies of a variety of immune-mediated diseases because of its efficacy and an acceptable safety profile, as most low-dose MTX associated toxicity has been described in case reports and relatively small case series [30].

However, although well-tolerated and mostly reversible, even a low-dose regimen of MTX can result in clinically significant toxicity with substantial death rates (about 25% according to Kivity's cohort study) [30]. The low-dose MTX associated severe adverse effects include major central nervous system complications [83], mucositis, pulmonary involvement, hepatotoxicity [84], and myelosuppression.

### 5.3. Is MTX Safe to the Pregnant and Fetuses?

As one of the lipid-soluble and low molecular weight drugs, MTX could be readily transferred across the placental membrane during pregnancy and adversely affect the fetus [85]. In addition, MTX might take longer time for elimination in fetal tissues [86].

Regarding pharmacogenetics, mutations caused by MTX lead to severe decrease of the expression of folate and nucleobase enzymes, which are critical for cellular homeostasis [87]. In practice, MTX affected formation of the blastocyst and caused dysmorphic features and neurologic defects in early pregnancy, leading to malformations in some cases [88]. Multiple congenital abnormalities have been observed after weekly MTX treatments at a 10 mg dose during the first 3 months of pregnancy [89], even fetal death [85]. Verberne et al. had reviewed cases of congenital anomalies after in utero exposure to MTX and proved that some congenital anomalies such as microcephaly, craniosynostosis, tetralogy of Fallot, pulmonary valve atresia, limb reduction defects, and syndactyly were truly part of the “fetal methotrexate syndrome” [90]. Administration of MTX in childhood might also cause manifestations including visual defect [91] and Smith–Magenis syndrome [43] among patients with specific mutations. Thus, special care should be taken with pregnant patients and children in particular.

### 5.4. The Risk Factors of MTX Associated Side Effects

The most common risk facts of MTX induced adverse effects are advanced age (age > 75 years) and underlying disease including renal and/or hepatic insufficiency and lung disease, especially patients with chronic hepatitis B and diabetes mellitus [92–96]. Patients with a history of alcohol intake might have a greater risk of liver fibrosis and hepatotoxicity caused by MTX administration, with >100 g alcohol consumption per week [97]. Also, preexisting hypoalbuminemia and past use of any of the DMARDs and proton-pump inhibitors have been described in studies to increase the incidence of MTX induced side effects [92, 94]. Moreover, taking drugs that may interact with MTX at the same time might also be dangerous; these drugs include salicylates, cotrimoxazole, chloramphenicol, sulfonamides, cyclosporine, and pyrimethamine [96]. Although no significant protective effect of folate supplementation on MTX related toxicity has been found [98], the folate deficiency is another reason for the side effects, based on clinical cases [30]. Heidari et al. found that MTX administration elevated kidney ROS levels, decreased tissue antioxidant capacity, increased lipid peroxidation, and depleted renal glutathione stores. Their research data indicate that MTX caused tissue damage and organ dysfunction through oxidative stress. Therefore, they proposed that patients with preexisting mitochondrial defects might be vulnerable to MTX induced renal injury [99].

The use of high-dose MTX (HD-MTX) is also the risk factor of adverse effects. MTX induced liver fibrosis is more likely to become morphologically evident with high cumulative doses, possibly largely exceeding 3000 to 4000 mg [97, 100]; and the side effects caused by omeprazole use in the past were found in cancer patients receiving HD-MTX treatment [94].

The distribution of MTX in vivo also plays a role in MTX related side effects. As MTX tends to accumulate in the extravascular compartment, patients with pleural effusion, ascites, and massive edema should get extra caution, due to the risk of toxicity from reabsorption of extravascular fluid [101].

Another noteworthy risk factor is UV. UV recall phenomenon, also known as MTX associated UV reactivation, has been reported [102, 103]. It is reactivity of sunburn areas within 3 to 10 days of the treatment with MTX [103, 104]. According to Adams and associates, this phenomenon might be due to the immune response by uncontrolled sunburn induced inflammation released by MTX [104]. Patients who previously suffered sunburns deserve more detailed monitoring when methotrexate is needed.

### 5.5. Is Folate Supplementation Necessary for Ophthalmic Patients?

To prevent MTX associated side effects, it is common to take folate [as either folic acid (FA) or folinic acid (FLA)] in clinic [46, 105, 106]. However, there is no consistent and evidence-based guideline for folate supplementation in ophthalmic patients.

Folate and folic acid play significant roles in the de novo synthesis of purines and thymidylate, which are required for DNA replication and repair [96]. Funk and associates found a significant reduction of circulating folate concentrations in 47% of patients receiving MTX treatment [107]. Patients treated with high-dose MTX (HD-MTX) got routine folate supplementation to reduce HD-MTX associated side effects [108–110]. After a systematic literature review of HD-MTX therapy and folate supplementation, Van der Beek et al. [111] found lower incidence of MTX associated adverse effects in regimens with higher cumulative doses and earlier administration of folate supplementation, in similar HD-MTX dosage studies. Folate supplementation in patients with low-dose methotrexate is also being studied. Ortiz et al. [105] had proved the protective effect of folate supplementation by conducting a Cochrane review including more than 600 patients taking low-dose MTX. Until now, folate supplementation had been proved to prevent and improve MTX associated effects including gastrointestinal, respiratory, and neurologic side effects [96, 112]. Mori et al. supported the protective effect by demonstrating that patients treated with low-dose MTX without folate supplements were significantly associated with the development of myelosuppression and pancytopenia [113].

However, Arabelovic and associates' preliminary study showed a significant increase of MTX dose needed [114], since folic acid fortification enriched cereal grain products were fully implemented in the USA and Canada [115]. This conveyed a message to us that high dose of folate supplementation might have influence on the efficacy of MTX.

Al-Dabagh et al. found that the reduction in efficacy of MTX cannot be ignored while folate supplementation did make a significant reduction in associated adverse effects [116]. Salim et al. declared the decreasing influence between the anti-inflammatory effect of MTX and folate supplementation, by carrying out a double-blind clinical trial [117]. Chladek et al. had conducted an open-label, two-way crossover study, supporting the opinion above [118]. Additionally, because of the unequal distribution of folic acid and MTX in organs and tissues [119], MTX discontinuation is more common for some MTX associated side effects in ophthalmic clinic [112], rather than higher dosage of folate supplementation.

There are no ophthalmic studies to demonstrate the protective effects of folic acid supplementation. Thus, although the folate supplementation is widely used among patients treated with low-dose MTX [120, 121], the necessity and standardized dosage of folate supplementation in specific patients [122], as well as the MTX-folate interaction, still warrant further studies.

## 6. Discussion

Methotrexate, as one of the alternative pharmacological steroid-sparing immunosuppressive agents, is becoming more and more popular as the preferred treatment in several autoimmune conditions requiring long-term immunosuppression [123]. Low-dose MTX has anti-inflammatory and immunomodulatory properties by increasing levels of intracellular and extracellular adenosine [124], which is the foundation of ophthalmic MTX treatment. The standardized and recommended administration of ophthalmic MTX treatment is once a week, starting with a dose of 7.5 mg and escalating every 4 to 8 weeks up to 25–30 mg/week when necessary [125, 126]. In patients with insufficient response to MTX alone, cyclosporin with or without azathioprine was added [127].

To avoid side effects, split doses of MTX administration and folate supplementation are gradually being used in ophthalmic clinic. Prescription of 5 to 10 mg of folate supplementation has a significant role in MTX safety [128], but the higher dosage is less applied, even with higher dose of MTX [129]. Prophylactic folate supplementation is not necessary in most patients [130]. There is also research to convey that 0.5 ml/100 g or above dosage of fish oil is as effective as folinic acid in therapeutic potential in preventing bone loss during MTX chemotherapy [131]. For some resistant and/or mortal adverse effects, the discontinuation of MTX will work instantly.

With the increasing long-term use of MTX, it is important to monitor patients' blood examination results, including blood routine and liver and renal functions. As pancytopenia can be a late manifestation [98], elevation of urea, creatinine, aminotransferases, and albumin as well as electrolytes disturbances may result in MTX associated liver and renal side effects [99]. Plasma MTX level is not a reliable predictor for adverse events in MTX therapy [132]. On the contrary, circulating folate levels and folate polyglutamate distribution change sensitively with MTX exposure and exogenous folate supply [133] and could be used as a biomarker of MTX efficacy [107]. It should be noted that as erythrocytes have a half-life of approximately 120 days, the results of blood examinations might reflect both pretreatment and posttreatment status, which need to be analyzed carefully [99].

Numerous studies had been conducted to prove that MTX could be used as a well-tolerated, safe, and effective first-line treatment. Hence, the MTX administration should not continue to be stigmatized as a “cancer drug,” or to be discouraged because of associated adverse effects. Contrarily, the indication and the routes of administration are about to gradually widen.

## Figures and Tables

**Table 1:** Summary of genes and their SNPs which might have possible clinical effects towards MTX.

	Gene		SNP(s)	Possible clinical effects
Transporting proteins	RFC1 [31–34]		G80A	Increasing/decreasing intracellular MTX level
	ABC family	ABCB1 [28, 29, 38]	C3435T	Affecting efficacy of MTX
			C1236T	Affecting the distribution of MTX and incidence of hematological toxicity
		ABCC1 [47, 48]	rs246240S	Association with MTX related toxicity
			rs3784862	
		ABCC2 [29, 45]	A2412G	Leading to accumulation of MTX to nephrotoxic levels
			G1249A	Association with MTX related gastrointestinal toxicity
			G1058A	Association with MTX related hepatotoxicity
		ABCC4 [28]	C934A	Association with MTX related hematological toxicity
Metabolizing enzymes	MTHFR [39–44]		C677T	Affecting the toxicity but not the efficacy by resulting in a lower enzyme activity; association with related nephrotoxicity, anemia, and neurologic side effects
			A1298C	
	ATIC [49–51]		C347G	Affecting efficacy and toxicity of MTX
	TYMS [52, 53]		5′-UTR 28-bp repeat	Affecting efficacy and toxicity of MTX
			3′-UTR 6-bp deletion	Affecting efficacy of MTX.
	GGH [46, 54, 55]		C452T	Affecting efficacy of MTX
			C401T	
	DHFR [29, 45]		T721A	Affecting efficacy of MTX
			C830T	
	MS [29, 45, 54]		A2756G	Association with MTX associated toxicity
	MTRR [29, 45, 51]		A66G	Association with MTX associated toxicity
